# Proteasome Inhibitors: Harnessing Proteostasis to Combat Disease

**DOI:** 10.3390/molecules25030671

**Published:** 2020-02-05

**Authors:** David J. Sherman, Jing Li

**Affiliations:** 1 Amgen Research, Amgen Inc., Thousand Oaks, CA 91320, USA; dsherm01@amgen.com; 2 Division of Biology and Biological Engineering, California Institute of Technology, Pasadena, CA 91125, USA; 3 Biochemical and Cellular Pharmacology, Genentech Inc., South San Francisco, CA 94080, USA

**Keywords:** proteostasis, proteasome, ubiquitin, immunoproteasome, unfolded protein response, Rpn11, Rpn13, p97, bortezomib, carfilzomib, ixazomib, oprozomib, marizomib, KZR-616, capzimin, RA190, RA183, KDT-11, RIP-1, NFE2L1/Nrf1

## Abstract

The proteasome is the central component of the main cellular protein degradation pathway. During the past four decades, the critical function of the proteasome in numerous physiological processes has been revealed, and proteasome activity has been linked to various human diseases. The proteasome prevents the accumulation of misfolded proteins, controls the cell cycle, and regulates the immune response, to name a few important roles for this macromolecular “machine.” As a therapeutic target, proteasome inhibitors have been approved for the treatment of multiple myeloma and mantle cell lymphoma. However, inability to sufficiently inhibit proteasome activity at tolerated doses has hampered efforts to expand the scope of proteasome inhibitor-based therapies. With emerging new modalities in myeloma, it might seem challenging to develop additional proteasome-based therapies. However, the constant development of new applications for proteasome inhibitors and deeper insights into the intricacies of protein homeostasis suggest that proteasome inhibitors might have novel therapeutic applications. Herein, we summarize the latest advances in proteasome inhibitor development and discuss the future of proteasome inhibitors and other proteasome-based therapies in combating human diseases.

## 1. Introduction

Cells have evolved intricate regulatory mechanisms to adjust their proteomes in response to intracellular and environmental conditions, allowing for healthy growth and survival [1]. Aberrancies in protein synthesis, folding, trafficking and degradation can lead to many human illnesses and diseases, including neurodegenerative disorders, cancers, and autoimmune diseases [2,3,4]. The link between protein homeostasis, or proteostasis, and human health have motivated expansive efforts over the past three decades to discover and develop modulators of the major mammalian protein degradation pathway: the ubiquitin-proteasome system (UPS) [5,6]. The UPS is involved in a range of essential cellular processes, including cell-cycle progression, antigen presentation, inflammation, apoptosis, DNA repair, signal transduction, and protein quality control [7,8]. For example, the UPS is central to the unfolded protein response (UPR), which is activated when unfolded or misassembled proteins accumulate in the endoplasmic reticulum (ER). The UPR leads to apoptosis if ER stress is not mitigated [9,10]. The UPS is also critical for regulating the transcription factor NF-κB, thereby affecting the inflammatory response in addition to apoptosis and oncogenesis [11,12]. Neurons rely heavily on the UPS for maintaining proteostasis because of their long lifespans and specialized architectures, and deficiencies in the UPS are hallmarks of neurodegeneration [13,14,15].

While the fundamental mechanistic and structural details of the proteasome are still active areas of investigation [16], the use of proteasome inhibitors in clinic has proven to be successful in treating cancer, specifically multiple myeloma (MM) and mantle cell lymphoma (MCL), with the number and types of disease indications growing [17,18,19,20]. However, there are considerable challenges with using proteasome inhibitors because of the critical role that the proteasome plays in fundamental cellular processes. Additionally, while proteasome inhibitors have been successfully employed to treat cancer, where inhibiting uncontrolled cell growth is important, there are other circumstances in which activating the proteasome could be advantageous, including in the treatment of neurodegeneration. Proteasome activation has also been found to delay retinal degeneration in mice [21]. There is a fine line between having too much and too little proteasome activity; in some circumstances, one might want more proteasome activity, while in others, it is better to have less. There also must be a balance between preserving enough proteasome activity for survival of most cells in the body and eliminating unwanted activity in specific cells types or organs. With the number of clinical trials involving proteasome inhibitors alone or in combination with other therapies growing, and diseases treated with proteasome inhibitors expanding, it is an exciting time to think about how to improve current proteasome inhibitors and novel strategies for developing new ones. In addition to highlighting the current state of proteasome inhibitors in laboratory and clinic, this review will offer perspectives on how to improve the efficacy of current proteasome inhibitors (alone or as combination therapies), directions for developing new UPS inhibitors (including for targets other than the proteasome itself), and fundamental research directions that will enhance proteasome inhibitor development and validation.

### 1.1. Proteasome: Structure and Function

#### 1.1.1. 26S Proteasome

**Figure 1:**
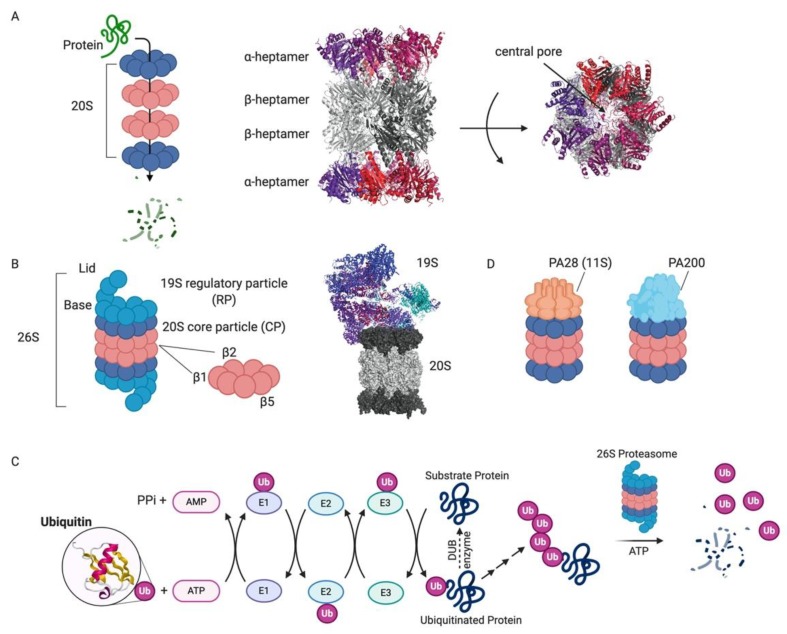
Proteasome and ubiquitin-mediated degradation [85]. (**A**) Schematic of substrate protein degradation by passage through the central pore of the four stacked heptameric rings that comprise the 20S proteasome *(left)*. The X-ray crystal structure of the yeast 20S proteasome (PDB 1RYP) shows the two capping α-heptameric rings (red/violet) and the middle two β-heptamers (grey; *middle)*. The central pore through which substrates are threaded is indicated in the top view *(right)*. (**B**) The 26S proteasome is the central component of the UPS. A cartoon depiction of the components of the 26S proteasome highlights the lid and base of the 19S RP in addition to the DUB Rpn11 and the catalytic β-rings *(left)*. The cryo-EM structure of the human 26S proteasome (PDB 6MSB) shows the structure of the 26S proteasome in a state competent for ubiquitinated substrate engagement. (**C**) Schematic of the ubiquitin-proteasome system (ubiquitin PDB 1UBQ). (**D**) Schematic of alternative 20S proteasome complexes with a heptameric PA28 cap or a monomeric PA200 cap.

The presence of a soluble ATP-dependent “proteolytic machine” was discovered in the 1970′s [22], and the identification and characterization of the proteasome and the role of ubiquitin in protein degradation emerged over the following 20 years [5,8,23,24]. The proteasome is a 2.4 megadalton multi-subunit enzyme complex of the conserved AAA+ (ATPase associated with various cellular activities) family [25]. The 26S proteasome degrades the majority (at least 80%) of proteins in the cytoplasm and nucleus of mammalian cells [26]. Proteasome substrates include misfolded or damaged proteins in addition to regulatory proteins that need to be degraded for proper cellular function. The central component of the 26S proteasome is the 28-subunit “core particle” (CP) comprising four stacked heptameric rings that form a donut-shape (referred to as the 20S proteasome). The middle of the donut creates an axial pore through which unfolded polypeptides are translocated (Figure 1A). The two outermost rings, formed by seven α-subunits, mediate interactions between the central pore and the cellular environment. The innermost rings are formed by seven β-subunits, and three of these subunits contain the proteasome’s enzymatic active sites. The N-terminal threonine of each enzymatic subunit serves as both the nucleophile and primary proton acceptor in the peptide bond cleavage reaction. Each active site has similar cleavage specificity to a known protease: β1 has caspase-like specificity, β2 has trypsin-like specificity, and β5 has chymotrypsin-like specificity. Despite the proclivity of each active site to cleave after certain amino acid types, the close proximity of the six sites facing the narrow axial pore allows for the processive degradation of a broad range of primary amino acid sequences into small peptides (~10 amino acids). These peptides can be further processed and used for antigen generation, or the amino acids can be recycled for new protein synthesis [27].

What prevents the proteasome from nonspecifically degrading cellular proteins? First, the active sites are sequestered within a deep pore, topologically preventing nonspecific proteolysis. The narrow radius of the pore prevents folded proteins from entering. Second, the CP itself is auto-inhibited by the N-terminal tails of the α-subunits [28], requiring regulatory factors to open the gates of the channel [29,30]. This allows for multiple regulatory checkpoints before a substrate is degraded. The main regulatory component of the 26S proteasome is the 19S regulatory particles (RP), also known as PA700, that caps either or both ends of the 20S CP (Figure 1B). The 19S RP is remarkably complex and comprises 19 stoichiometric subunits and many sub-stoichiometric interacting proteins [8,31]. UPS substrates must pass through this complex in order to be degraded, as the 19S RP opens the gates of the central channel of the 20S CP.

Prior to proteasomal degradation, proteins must be properly “tagged” and targeted to the proteasome. A cascade of enzymes, known as E1, E2, and E3 enzymes, are responsible for this “tagging” (Figure 1C). These enzymes catalyze isopeptide bond formation between the C-terminus of an 8 kDa protein called ubiquitin and the lysine side chain of the substrate (“acceptor”) protein. Ubiquitin chains can be elaborated by attaching additional ubiquitin moieties to any of its seven lysine residues, or the N-terminal amino group. At least four ubiquitin molecules are required to tag a substrate for proteasomal degradation [32], although there are some reported exceptions to this rule [19]. The large diversity of E3 enzymes in mammalian cells confers substrate specificity for ubiquitin conjugation. Ubiquitin chains are often expanded to form chains of different sizes and with branches at different lysine residues within the ubiquitin protein. Poly-ubiquitin chains, especially with K48 linkages, selectively mark proteins for degradation by the 26S proteasome [8]. Therefore, this complex pathway diversifies the range of substrates for the proteasome and enhances the regulation of proteins destined for degradation [33].

The 19S RP is divided into two biochemically separable sub-complexes: the base and the lid [34]. The distal lid contains at least nine proteins that are responsible for ubiquitin binding and removal. One core component of the lid is a Jab1/MPN domain-associated metalloisopeptidase (JAMM) domain-containing protein Rpn11 (*POH1*/*PSMD14*) that couples deubiquitination with degradation (Figure 1B) [35,36]. The development of Rpn11 inhibitors might have the ability to expand effective UPS inhibitors in the clinic, as discussed below [37,38,39,40]. The base of the 19S RP includes a ring-shaped heterohexamer of AAA+ ATPases that engage and unfold substrate polypeptides to allow for their translocation into the proteolytic pore of the 20S CP [8,16,31]. The ATPases couple ATP hydrolysis with the mechanical opening of the gates in the α-subunits of the 20S core.

#### 1.1.2. Alternative Proteasome Complexes

In addition to the 26S proteasome, there are several other 20S complexes that catalyze ubiquitin- and ATP-independent degradation and serve a variety of roles in cells. These complexes include proteasome “activators” that open the gates of the central pore of the 20S CP, as the CP itself has very limited basal activity because of its auto-inhibition. Notably, the alternative 20S caps do not have polypeptide unfoldase activities. One such complex contains the 11S activator (PA28) in place of the 19S RP (Figure 1D). The 11S activator is mostly found in higher eukaryotes and is induced by interferon gamma, although there is basal expression in all tissues [19,41]. There are three different PA28 subunits (α, β and γ) that can form two kinds of heptameric barrel-like structures (either with PA28αβ or PA28γ). C-terminal motifs in PA28 subunits bind the α-subunits of the 20S CP and contain an internal activation loop that is involved in pore opening [42,43,44]. PA28αβ plays a role in antigen processing and MHC Class I antigen presentation [45,46,47]. Additionally, PA28γ has been shown to promote the degradation of cell cycle regulatory proteins such as p21 and SRC-3 [48,49]. Blm10/PA200 is another ubiquitin- and ATP-independent proteasome activator that is mostly found in the nucleus and has been implicated in such processes as DNA repair, mitochondrial function, and spermatogenesis (Figure 1D) [50,51,52,53]. Unlike the 11S activator, Blm10/PA200 is a monomeric ~250 kDa protein, conserved from yeast to higher eukaryotes, that forms a dome around the entrance to the 20S pore and makes extensive contacts with the α-subunits of the 20S CP. Blm10/PA200 has been found to stimulate the hydrolysis of small peptides and the unstructured tau protein in vitro [54]. Cumulatively, label-free quantitative proteomic analysis indicates that less than 10% of total cellular proteasomes are bound to ubiquitin-independent activators [55]; however, recruitment of PA28γ and PA200 to proteasomes is an unexpected consequence of catalytic inhibition, suggesting that their roles in cell biology might have clinical significance, such as in resistance to proteasome inhibition [56]. Additionally, the biological roles and structural characterization of hybrid proteasome complexes containing one 19S RP and one alternative cap raise the possibility that alternative proteasome structures, which can be “tuned” to specific cellular and environmental contexts, might be important therapeutic targets [57,58,59,60].

The presence of ATP- and ubiquitin-independent roles for the proteasome highlights the fundamental question of how substrates are specifically and selectively targeted for degradation. To be degraded, there must be a proteasome targeting mechanism, and ubiquitin solves that problem for UPS substrates. What about substrates that are degraded in a ubiquitin-independent manner? This is largely an unanswered question, but it is clear that proteins degraded in a ubiquitin-independent manner must be intrinsically disordered or contain disordered regions in order to fit into the proteolytic core [13]. It is estimated that up to 41% of eukaryotic proteins have intrinsically disordered regions, suggesting, at least in principle, that a significant portion of the proteome can be degraded in a ubiquitin-independent manner [61]. Well-characterized proteins that are degraded in a ubiquitin-independent manner include the yeast transcription factor Rpn4, thymidylate synthase, and ornithine decarboxylase [62]. Interestingly, in the case of ornithine decarboxylase, proteasomal degradation requires ATP and is accelerated by antizyme I; additionally, ubiquitinated substrates or free polyubiquitin chains compete for its proteasomal recognition [63,64]. This suggests that the same machinery responsible for ubiquitin-dependent degradation can also degrade proteins in a ubiquitin-independent manner. Discovering new substrates of ubiquitin-independent proteasome degradation pathways, and mechanistic details of these pathways, will be informative for developing inhibitors that block degradation of particular proteins important in human health and disease. Conversely, activating proteasomal degradation of intrinsically disordered proteins could be useful for treating neurodegenerative diseases such as Alzheimer’s disease, Parkinson’s disease, and amyotrophic lateral sclerosis [13].

#### 1.1.3. Immunoproteasome

During oxidative stress or in response to proinflammatory cytokines (e.g., interferon-gamma), cells produce specialized proteasome assemblies called immunoproteasomes [65]. Antigen-presenting cells have greater basal expression levels of immunoproteasome subunits. Immunoproteasomes are large proteasome complexes that are nearly identical to the constitutive proteasome except for different catalytic β-subunits (β1i, β2i, and β5i) that share high sequence homology with the associated constitutive proteasome β-subunits [65,66] (Figure 1). Interestingly, the immunoproteasome has a different substrate specificity than the constitutive proteasome and favors production of peptides that terminate in basic or hydrophobic amino acids [67,68,69,70,71]. Therefore, immunoproteasomes enhance ligand generation for MHC Class I molecules, allowing for surveillance by CD8^+^ T lymphocytes. Immunoproteasome formation following cytokine induction is rapid, cooperative, and can be transient [72,73,74]. Originally thought to be strictly involved in antigen presentation, immunoproteasomes have also been implicated in other cellular processes, including T cell polarization and cytokine production by macrophages [66]. Immunoproteasomes generate different peptides than constitutive proteasomes because they contain slightly different substrate binding pockets [75]. There is considerable effort to generate immunoproteasome-specific inhibitors, as immunoproteasome complexes appear to be present at low levels normally but higher levels in certain cancers, inflammatory diseases, and autoimmune diseases. Therefore, immunoproteasome inhibitors can be more selective and have fewer toxic side effects than constitutive proteasome inhibitors [76,77]. KZR-616, the only immunoproteasome inhibitor that has advanced to clinical trials, is described below. Based on the reversible epoxyketone β5i inhibitor ONX0914, the compound shows minimal cross-reactivity with the constitutive β5 subunit [17]. Advances in understanding the structural differences between the constitutive proteasome and the immunoproteasome will lead to the development of better, more specific and potent immunoproteasome inhibitors [66,75,78,79]. In addition to the immunoproteasome, it should be noted that there is a thymus-specific proteasome (thymoproteasome) that is involved in thymic positive selection and the development of CD8^+^ T lymphocytes [72,80]. The thymoproteasome incorporates a different β5 subunit, denoted β5t, and thymoproteasome-expressing cells display a unique set of antigenic peptides [72,80,81]. Finally, the testes-specific spermatoproteasome contains unique 20S proteasomal subunits in addition to some immunoproteasome subunits [82]. The PA200 activator is also abundant in testes and plays a role in spermatogenesis [83,84].

## 2. Proteasome Inhibitors

Scientists have been using proteasome inhibitors for many years to interrogate the biological function of the proteasome and its involvement in various cellular processes. The essential role of the proteasome in cell biology makes proteasome inhibition challenging to manage: one wants to achieve enough inhibition to kill diseased cells that rely heavily on the proteasome but not healthy cells. Complete proteasome inhibition is toxic. This was the early rationale for developing proteasome inhibitors to treat neoplastic cells that proliferate rapidly and have aberrant cell cycle checkpoint regulation [86]. Proteasome inhibitors are especially effective at treating hematological cancers because of the high dependence of these cancer cells on proteostasis mechanisms (i.e., they have a high secretory load) [17,87,88,89]. These cancer cells rely heavily on several mechanisms collectively called ER-associated degradation (ERAD), which dislocate aberrantly folded secretory and membrane proteins from the ER to the cytosol, where they are degraded by the proteasome [90]. We discuss the range of proteasome inhibitors used in the clinic for treating hematological cancers, and those under investigation in the lab for treating other forms of cancer and diseases.

### 2.1. Structural Characteristics of Proteasome Inhibitors

The proteasome inhibitors discussed in this review are largely covalent electrophilic inhibitors that react with the active-site threonine residues of the proteasome. The first proteasome inhibitors were analogs of serine protease inhibitors: hydrophobic peptide aldehydes that mimicked substrates of the β5 active site. Peptide aldehydes react with the nucleophilic hydroxyl group of threonine to form reversible hemiacetal adducts. One of the early aldehyde inhibitors, MG132, is a tool compound that is still used by many researchers today. However, MG132 has off-target effects in cells, including the inhibition of cathepsin B and calpains [7]. As a consequence of its structure, it is also quickly oxidized. In the years following the discovery of MG132, there were major advances in the development, optimization, and applications of new proteasome inhibitor structural scaffolds. Other structural classes, such as peptide boronates (e.g., bortezomib and ixazomib) and peptide epoxyketones (e.g., carfilzomib, oprozomib, and immunoproteasome-specific inhibitors ONX0914 and KZR-616), have had great success in the clinic, in part because the active-site adducts that they form are stabilized by specific interactions with the threonine amino group. For example, peptide boronates have an additional stabilizing hydrogen bonding interaction, and epoxyketones form stable morpholine adducts [18]. These additional interactions greatly reduce the off-rates of these inhibitors, making some of them essentially irreversible under physiological-treatment conditions. While most clinical proteasome inhibitors are covalent peptidic molecules, we also describe a nonpeptidic inhibitor, marizomib, which contains a reactive β-lactone that forms a tetrahydrofuran ring with the active-site threonine [18]. While more specific than peptide aldehydes, β-lactone inhibitors are less specific than epoxyketones. Nonpeptidic inhibitors were also developed for the immunoproteasome with the aim of improving metabolic stability and bioavailability, which is a general liability of peptidic backbones [91,92,93]. The lead compound from a virtual-screening effort showed excellent selectivity for β5i compared to the constitutive ®5 subunit [91]. However, the cell-based activity of this compound is much weaker than that of ONX0914 [91]. More efforts are needed to improve the compound’s potency. Efforts to screen for novel nonpeptidic inhibitors of the constitutive proteasome and immunoproteasome could result in molecules with improved pharmacokinetics and broader therapeutic potential. However, with significant interest in improving peptidic therapeutics [94], it is unclear which class of molecule is ultimately most efficacious in the proteasome inhibitor space.

The enzymatic mechanism of the 20S proteasome has prompted certain electrophilic structural classes of inhibitors to stand out, and the peptidic nature of many 20S inhibitors resembles natural proteasome substrates. In addition to inhibiting the 20S proteasome, there are multiple new inhibitors that target other UPS components, conferring unique phenotypes and toxicity profiles when used in preclinical studies. These inhibitors also represent more diverse structural classes than 20S inhibitors because of their mechanisms, opening the door to other therapeutic indications. Although proteasome inhibitors have seen the most success to date in treating MM and MCL, the list of inhibitors being tested, either alone or in combination with other drugs, to treat solid tumors and other diseases is long and growing.

### 2.2. Proteasome Inhibitors in Clinic

#### 2.2.1. Bortezomib (Velcade)

**Figure 2:**
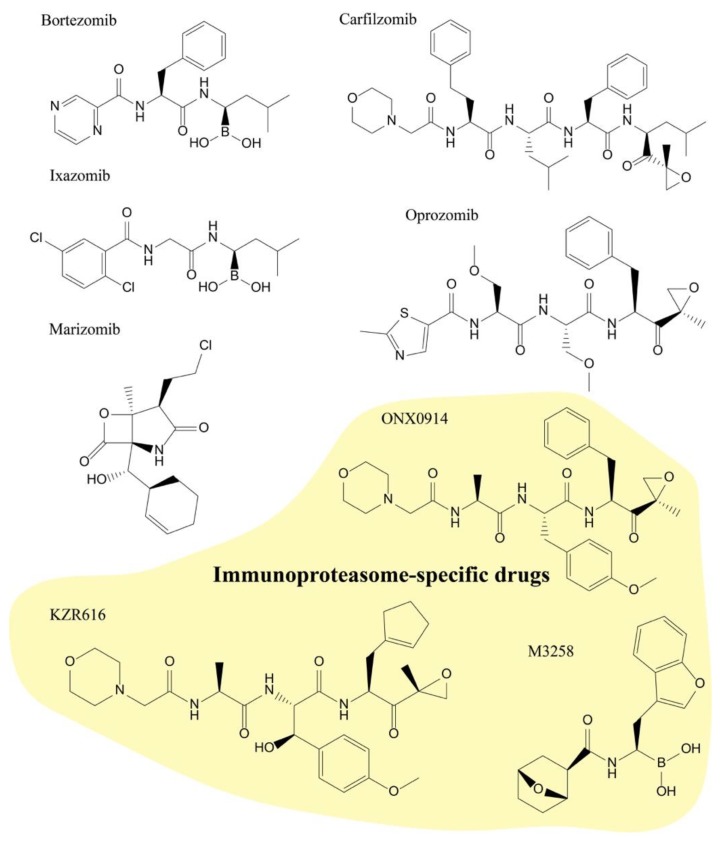
Clinical proteasome and immunoproteasome inhibitors. Chemical structures of clinically used proteasome and immunoproteasome inhibitors. For more information, see Table 1 and Table 2.

Bortezomib was the first clinically approved proteasome inhibitor and is now indicated for the treatment of newly diagnosed multiple myeloma (NDMM) and relapsed MCL (Figure 2). The introduction of bortezomib into the clinic was a milestone in the treatment of MM, prolonging progression-free survival, and improving overall survival in patients with NDMM [95,96,97]. Bortezomib is a slowly reversible peptide boronate inhibitor that binds the catalytic site of the 20S proteasome, enabling the potent inhibition of the chymotrypsin-like activity of the β5 subunits (IC_50_, 7 nM; Table 1) [98]. Bortezomib also inhibits the trypsin-like activity of the β2 subunits and caspase-like activity of the β1 subunits at high concentrations, but these inhibitory activities are much less potent compared to its inhibition of the β5 sites [98]. Since its FDA approval in 2003 for refractory disease, bortezomib has become the frontline therapy for MM patients [99]. At therapeutic doses, bortezomib inhibits approximately 65% of β5 activity in patient whole blood lysate, which effectively induces apoptosis in myeloma cells without causing toxicity to nontransformed cells [89,100]. Given the essential role of the proteasome in all cells, it is critical to understand the mechanism of bortezomib specificity for MM cells. Physiologically, MM cells have elevated NF-κB activity, which is vital for their survival and proliferation [101,102]. Proteasome inhibitors block the NF-κB pathway by inhibiting the proteasomal degradation of the NF-κB inhibitor IκB, therefore enhancing the therapeutic efficacy of conventional treatments such as dexamethasone and lenalidomide [103]. This partially explains the reason why bortezomib specifically targets transformed MM. However, it is unlikely to be bortezomib’s critical mechanism of action because other NF-κB pathway inhibitors, like IκB kinase inhibitors, cannot kill MM cells as effectively as bortezomib [104]. An additional explanation for the sensitivity of MM cells to bortezomib is the cells’ low tolerance for proteotoxic stress. Bortezomib inhibits the primary protein-recycling machinery in cells, leading to the accumulation of misfolded proteins and UPR induction. A sustained UPR signal eventually triggers irreversible cell death [89]. MM cells produce large quantities of antibodies and rely heavily on the protective UPR. A brief exposure to the proteasome inhibitor can tip the balance to a terminal UPR, which commits MM cells to an apoptotic fate [88]. Therefore, MM cells appear to be prone to a cytotoxic bortezomib-induced terminal UPR.

Despite the fact that bortezomib is a successful therapy compared to conventional chemotherapeutic agents, it is not a panacea. It has several limitations. First, it is not uncommon for resistance and relapse to occur in patients who initially respond well to it. Several factors were found to contribute to bortezomib resistance. For example, mutations in the β5 subunits were identified in bortezomib-resistant MM cell lines [107,108]. These mutations, such as A108T and G322A, change the bortezomib binding pocket and affect β5–bortezomib interaction. Second, there are changes in cellular proteasome subunit composition and expression upon inhibition [109]. In addition to the changes in the proteasome itself, global changes in the transcriptome, such as increased expression of antiapoptotic proteins and decreased expression of proapoptotic proteins, also affect the sensitivity of MM cells to bortezomib [110,111]. Environmental factors, such as the bone-marrow microenvironment, also play a role in supporting bortezomib resistance [112]. In addition to acquired resistance, a significant issue limiting the usage of bortezomib is its toxicity towards the peripheral nervous system [113]. Patients treated with bortezomib can develop peripheral neuropathy (PN), which results in a painful burning sensation, numbness, and tingling in the extremities. PN is the primary cause of dose reduction or discontinuation among patients. Lastly, despite its efficacy in treating hematologic malignancies such as MM and MCL, bortezomib has had little success in clinical trials for treating solid tumors because of its rapid clearance from the blood and poor tissue penetration [114].

#### 2.2.2. Carfilzomib (Kyprolis)

The success of bortezomib inspired researchers to hunt for a new generation of proteasome inhibitors to overcome its shortcomings. In 2012, the FDA approved the first second-generation proteasome inhibitor, carfilzomib (Kyprolis). Carfilzomib is an epoxyketone with a structure based on the natural product epoxomicin (Figure 2) [115]. It inhibits the chymotryptic activity of the β5 subunit with high potency and specificity, with an IC_50_ value of ~6 nM (Table 1) [98]. Unlike bortezomib, carfilzomib is an irreversible inhibitor that forms a dual covalent bond with active site Thr1 in the β5 subunits [116]. Compared to bortezomib, carfilzomib significantly prolonged the progression-free survival in patients with refractory and relapsed MM [117,118]. Combination therapy with carfilzomib and immunomodulatory drugs for NDMM was also evaluated in multiple clinical trials. This treatment regimen was shown to be efficacious with a favorable safety profile in NDMM [119]. The major adverse event of bortezomib, peripheral neuropathy, was found to be less severe in patients receiving carfilzomib treatment [118]. Across several clinical trials, 18.1% of phase 1–3 MM patients treated with carfilzomib experienced adverse cardiovascular events of all grades [120]. These complications can be managed by a combination of basic risk assessments, hydration, cardiovascular monitoring, intervention (such as reducing the carfilzomib dose), and patient education to notice cardiovascular symptoms and regularly monitor blood pressure [121]. Clinical guidelines were developed in collaboration with cardiologists to minimize the risk of cardiovascular events in patients treated with carfilzomib [122]. Despite the improvements of carfilzomib over first-generation proteasome inhibitors, it still has to be administered intravenously, and has demonstrated limited efficacy in patients with solid tumors [123]. The lack of efficacy is potentially due to the different physiology in solid tumors, and the pharmacokinetics of the “omib” drugs [89].

#### 2.2.3. Ixazomib (Ninlaro)

In 2015, a third proteasome inhibitor, ixazomib (Ninlaro), was approved by the FDA to treat relapsed/refractory MM [124]. It was developed as a second-generation peptide boronic acid-based proteasome inhibitor and, notably, was the first orally available proteasome inhibitor (Figure 2) [105]. It is administered as a boronic ester prodrug that rapidly hydrolyzes to form the active inhibitor [105]. Although both bortezomib and ixazomib are reversible inhibitors from the same class with similar potencies, ixazomib has a much faster off-rate for proteasome binding than bortezomib (18 vs. 110 min *t*_1/2_; Table 1) [105,125]. When bortezomib is bound to the proteasome in red blood cells, its slow dissociation rate limits the drug distribution. Ixazomib was developed to overcome this limitation. Ixazomib’s fast off-rate allows for it to associate and dissociate with multiple proteasomes, allowing for its accessibility to more cells and larger tissue distribution [105].

### 2.3. Proteasome Inhibitors in Clinical Trials

#### 2.3.1. Marizomib

Marizomib is a natural product isolated from the marine actinomycete *Salinispora tropica*, and the only nonpeptidic proteasome inhibitor currently in clinical trials (Figure 2) [126,127]. The combination of marizomib, pomalidomide, and low-dose dexamethasone has demonstrated promising activity in heavily pretreated, high-risk relapsed/refractory multiple-myeloma patients [126]. Marizomib is an orally available drug that belongs to the β-lactone chemical family and irreversibly inhibits the activity of all catalytic β subunits [128,129]. It is one of the most potent inhibitors against the proteasome’s chymotryptic activity, with an IC_50_ value of ~3 nM (Table 1) [98,130]. Marizomib was demonstrated to treat patients with relapsed and/or refractory MM, either alone or in combination with other drugs, without some of the side effects (e.g., peripheral neuropathy) of other proteasome inhibitors [126,131]. It is also the smallest proteasome inhibitor identified to date and, likely because of its more lipophilic structure, the only proteasome inhibitor in clinical trials that can cross the blood–brain barrier [132]. These unique properties make marizomib an attractive candidate for the treatment of central-nervous-system (CNS) malignancies. Marizomib was found to induce caspase 9-dependent cell death in glioblastoma (GBM) cells and mouse models [133]. Currently, it is in clinical trials for treating newly diagnosed GBM [134].

#### 2.3.2. Oprozomib

Oprozomib is the result of efforts to develop orally available epoxyketone proteasome inhibitors (Figure 2) [106,135]. It belongs to the same molecular family as carfilzomib and irreversibly inhibits the β5 subunit (Table 1) [106]. It is in clinical trials for treating newly diagnosed and relapsed/refractory MM. The trials indicate that oprozomib shows encouraging activity in both relapsed/refractory and newly diagnosed MM. It could become an important MM therapy if the concerns regarding its gastrointestinal tolerability are addressed [136,137,138,139]. Oprozomib has also been evaluated for treating solid tumors, but unfortunately no valid treatment efficacy has been observed so far [140]. However, considering that the patients in the ongoing clinical study were pretreated with other therapies and have advanced cancers, the efficacy of oprozomib for treating earlier stages of cancer is yet to be determined [140].

#### 2.3.3. KZR-616

KZR-616 is the only immunoproteasome inhibitor that has advanced to the stage of clinical trials [141]. It was developed on the basis of ONX0914, an epoxyketone that selectively inhibits the β5i subunit with an IC_50_ of ~39 nM (Figure 2 and Table 2) [142]. Unlike constitutive β5 inhibitors, such as bortezomib and carfilzomib, it reduces the production of proinflammatory cytokines and cell-surface MHC Class I expression without apoptotic effects [142]. ONX0914 has shown efficacy in animal models of autoimmune disorders without affecting normal immune function [143,144]. Compared to ONX0914, KZR-616 has improved solubility and shows better efficacy in mouse models of antibody-induced arthritis [141]. It is in clinical testing to treat autoimmune diseases [141], expanding the indications of proteasome inhibitors beyond cancer. Notably, a second immunoproteasome inhibitor, M3258, targeting β5i was disclosed and will be tested in combination with dexamethasone in an upcoming phase I clinical trial for treating MM [145].

**Table 2:** Immunoproteasome inhibitors.

Name	Type	IC_50_ (nM) Against Immuno- and Constitutive-Proteasome [141]					
		LMP7 (β5i)	β5	LMP2 (β1i)	β1	MECL-1 (β2i)	β2
ONX0914	Epoxyketone	39	422	287	>12700	902	927
KZR-616	Epoxyketone	39	688	131	>10600	623	604

### 2.4. Proteasome Inhibitors in Lab

The history of proteasome inhibitor development teaches us two critical lessons: (i) MM is a starting point but not an end point for proteasome inhibitor-based therapies, and there are more therapeutic areas worth exploring; and (ii) despite the tremendous patient benefits conferred by proteasome inhibitors over the last decade, there are opportunities to expand their utility. There is sustained enthusiasm for identifying other mechanisms to inhibit the proteasome because: (i) inhibition of the 20S core particle by ‘omibs’ may not be the best route to inhibit the proteasome; (ii) other mechanisms of proteasome inhibition may provide benefits to patients who are refractory to bortezomib and carfilzomib; (iii) other mechanisms of proteasome inhibition, when combined with carfilzomib, may yield synergistic effects that provide even more dramatic progression-free survival; and (iv) the improved potency and selectivity of carfilzomib and potentially other classes of proteasome inhibitors may allow for expansion into other hematological malignancies and solid tumors. Therefore, we describe alternative strategies to develop non-conventional proteasome inhibitors in the lab with the potential for use as therapeutics in the future.

#### 2.4.1. Block the Removal of Ubiquitin Chains from Substrates: Rpn11 Inhibitors

**Figure 3:**
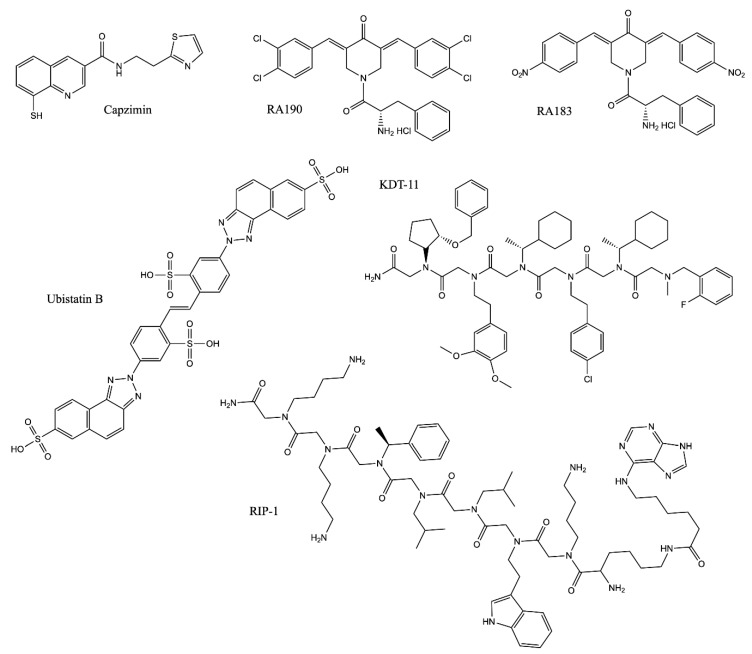
Structures of non-20S UPS inhibitors. For more information, see Table 3.

Rpn11 is an essential subunit of the 19S RP, which removes the polyubiquitin (polyUb) chains from substrates prior to degradation (Figure 1B) [35,146]. The active site is located within the JAMM domain of Rpn11, which contains a catalytic zinc ion coordinated by two histidine residues and an aspartate [147,148]. Importantly, substrate deubiquitination by Rpn11 is essential for degradation, to which it is tightly coupled [35,146]. Inactivation of the Rpn11 enzymatic active site by mutations of the zinc-coordinating histidine residues results in a severe decrease in substrate degradation in cells [33,149]. An HTS campaign with more than 300,000 compounds was conducted to discover Rpn11 inhibitors and led to the discovery of a metal-binding pharmacophore with a moderate IC_50_ of 2.5 µM [40,150]. Further optimization of this hit compound yielded capzimin, a more specific inhibitor with a biochemical IC_50_ of ~300 nM against Rpn11 (Table 3 and Figure 3) [40]. Capzimin blocks proteasome function by inhibiting the deubiquitinase activity of Rpn11, which causes accumulation of polyubiquitinated proteins, triggers the UPR, and blocks cell proliferation [40]. Capzimin is active against multiple cancer cell lines, including bortezomib-resistant cells [40]. In addition to capzimin, natural products such as thiolutin and gliotoxin were also identified as active inhibitors of JAMM proteases, including Rpn11. These compounds are active in cell culture and inhibit proteasome function by chelating the zinc ion in the Rpn11 active site [38,39]. However, these compounds are less specific towards Rpn11 than capzimin. Capzimin and other natural compounds need to be further optimized to improve the potency and specificity and to gain more drug-like properties.

#### 2.4.2. Block the Interaction between Substrate and the Proteasome

Rpn11 is not the only potential drug target in the 19S RP. The first reported inhibitor of the 19S RP was ubistatin, which blocks the binding of ubiquitin chains to their receptors [151]. Ubistatins A and B compete for the polyUb receptor Rpn10 and the shuttle protein Rad23 for their interactions with polyUb chains (Table 3 and Figure 3) [151]. As a result, ubistatins inhibit protein degradation in vitro [151]. More recently, a structural study revealed an interaction between ubistatin and ubiquitin. Ubistatin directly interacts with a hydrophobic patch and the surrounding basic/polar residues on the ubiquitin surface. Ubistatin shows a strong preference for K48 linkages over K11 and K63 linkages due to differences in the conformation and distribution of the hydrophobic patches on the surfaces [152]. The major challenge to further develop ubistatins as therapeutics is their negative charge, which precludes efficient membrane penetration, thereby limiting their potency in cells.

Another polyUb receptor in the 19S RP, Rpn13, is an attractive target for the development of alternative proteasome inhibitors [39]. Rpn13 and the deubiquitinating enzyme that it activates, Uch37, have been found to be important for cell cycle progression in cell culture [153], and increased expression of the gene encoding Rpn13 (*ADRM1*) has been identified in several forms of cancer [154,155,156,157]. Multiple efforts have been put forth to look for Rpn13 inhibitors. RA190, a bis-benzylidine piperidone, was the first identified inhibitor of Rpn13. It was believed to covalently interact with Cys88 of Rpn13 and block the binding of polyUb substrates (Table 3) [158]. RA190 induces the accumulation of polyUb proteins, causes ER stress, and stabilizes proteasome substrates, including p53 [158]. It showed anti-tumor effects in mouse models for MM and ovarian cancer [158]. A subsequent study suggests that RA190 has two synergistic mechanisms of action: targeting Rpn13 when it is not bound to the proteasome (at Cys88), and inactivating Uch37 at the proteasome [159]. Recently, an improved compound with higher potency, RA183, was developed and tested in mice [160]. Similar to RA190, RA183 is an irreversible inhibitor targeting Cys88 of Rpn13 and inhibits tumor growth in a xenograft mouse model of ovarian cancer (Table 3) [160]. In addition to RA190/RA183, a peptoid inhibitor, KDT-11, was identified as a Rpn13 inhibitor that binds with a modest K_d_ of 1.7 µM (Table 3) [161]. KDT-11 causes ubiquitin accumulation and acts synergistically with bortezomib in treating MM cells. KDT-11 is a reversible inhibitor that does not compete with RA190, indicating a different binding mechanism [161]. It should be noted that “enones” like RA190 have been described as “pan-assay interference compounds,” or PAINS, which raises concern about their specificity [162]. However, demonstrated examples of ways to circumvent the specificity problem with thiol-reactive compounds are described below (Section 3.2.2). Irrespective of the type of inhibitor, further support for inhibiting Rpn13 comes from the recent development of a proteolysis-targeting chimeric molecule (PROTAC), WL40, that fuses RA190 with thalidomide, targeting Rpn13 for degradation [163]. This molecule successfully inhibits MM cell growth in a mouse xenograft model. In fact, the molecule shows improved MM cell death compared to Rpn13 inhibitors. This presents a conundrum, though, because the PROTAC works by targeting Rpn13 for proteasomal degradation, but the effect of the drug is to hamper proteasomal degradation. Nonetheless, this proof-of-principle study shows that there could be some benefit to degrading a proteasomal or proteasome-associated target rather than functionally hindering it. There is a lot of excitement surrounding PROTACS like WL40 and their uses in selectively degrading proteins by linking a ubiquitin ligase to a particular target; the advantages and disadvantages of this strategy are reviewed elsewhere [164,165,166].

**Table 3:** Inhibitors of non-20S UPS components.

Name	Target	IC_50_/K_d_	Mechanism
Capzimin	Rpn11	0.3 µM (In vitro Rpn11 assay); 0.6 µM (Ub^G76V^-GFP degradation assay in cells)	Chelating the zinc ion in Rpn11, inactivating its DUB activity [40]
Ubistatin B	polyUb	10 µM (CFTR ubiquitination assay in cells)	Binding to the polyUb chain and blocking the substrate/Ub receptor interaction [151,152]
RA190	Rpn13	Not Applicable	Covalently binding to cysteine residue 88 (Cys88) of Rpn13 when it is not bound to the proteasome and Uch37 at the proteasome [158,159,160]
RA183			
KDT-11	Rpn13	1.7 µM (K_d_)	Peptoid ligand binding to Rpn13 [161]
RIP-1	Rpt4	3.0 µM (proteasome-mediated stripping of the Gal4-VP16 protein from DNA in vitro)	Inhibiting the protein unfolding activity of the 19S RP [167,168]

#### 2.4.3. Block Substrate Translocation into the 20S CP: Proteasome ATPase inhibitors

The third potential target in the 19S RP is the proteasomal ATPase, which provides the energy to power substrate translocation into the 20S CP and therefore facilitates protein degradation. RIP-1 was the first identified peptoid inhibitor targeting Rpt4 (*PSMC6*), a subunit of the proteasome ATPase [167,168]. RIP-1 biochemically blocks the protein unfolding activity of the 19S RP and inhibits turnover of the proteasome substrate p27 in cells (Table 3 and Figure 3) [167]. However, since the discovery of RIP-1 in 2007, there have been no follow-up studies to further understand the biological effects of this inhibitor. It will be interesting to understand its selectivity towards the proteasomal ATPase, given the abundance and similarity of ATPases in cells.

## 3. Perspective on the Use of Proteasome Inhibitors in Clinic

The initial development and clinical success of 20S proteasome inhibitors validated the maintenance of proteostasis as a therapeutic approach for treating human diseases. Proteasome inhibitors have revolutionized the treatment of MM, greatly extending patients’ lives. However, part of the success of proteasome inhibitors thus far is attributed to unique characteristics of transformed plasma cells. Additionally, the development of resistance and the toxicity profiles of currently used inhibitors cast doubts over the future utility of proteasome inhibition. The essential role of the proteasome in cell biology makes it difficult to envisage proteasome inhibition as a single therapeutic strategy for treating a range of human illnesses. To use proteasome inhibition to its full potential, it is critical to develop deeper understandings of the biological pathways affected by proteasome inhibition and strategies to confer inhibitor specificity to cell and proteasome types. This includes targeting other components of the UPS than the 20S proteasome itself. It also includes understanding mechanisms of proteasome resistance and trying to develop ways to stop resistance from developing.

### 3.1. Combatting Proteasome Inhibitor Resistance

Cancer cells are often described as resilient cells that continue growing despite efforts to stop them. True to the description, there have been multiple observed mechanisms of resistance to proteasome inhibitors in the lab and the clinic. Combatting these resistance mechanisms can serve as a potentially useful strategy for making proteasome inhibitors more effective for treating blood-based cancers and for expanding their profiles to treat solid tumors. Combination therapies comprised of a proteasome inhibitor and an inhibitor targeting a resistance mechanism is one example of a strategy that can be employed, much like amoxicillin and clavulanate are often used together to treat bacterial infections. Mechanisms of resistance to proteasome inhibitors (especially bortezomib) seen in the lab or clinic include, but are not limited to mutations in *PSMB5* (see Section 2.2.1), aberrant expression of UPS pathway components [169,170,171], induction of drug efflux from cells, and activation of signaling cascades that promote cell survival [172]. An early rationale for administering combination therapies to treat cancer was that drugs with nonoverlapping mechanisms would reduce the chances of developing therapeutic resistance [173]. The combination-therapy strategy showed promise for treating *BRAF*^V600E^ colorectal cancers [174]. *BRAF*^V600E^ melanomas, which have low expression of EGFR, are treatable with RAF inhibitors (e.g., vemurafenib); however, *BRAF*^V600E^ colorectal cancers adapt to RAF inhibitors through compensatory activation of the EGFR and MAPK signaling pathways [175,176]. An understanding of this biology paved the way for the encouraging results of a clinical trial for combination BRAF–EGFR–MEK inhibitor therapy for treating *BRAF*^V600E^ colorectal-cancer patients [174]. Although more has to be done to optimize the treatment regimen, this example highlights the power of understanding compensatory-resistance mechanisms for improving cancer therapies.

An interesting mechanism of resistance to sublethal proteasome inhibition involves activation of ER resident transcription factor Nrf1/NFE2L1. Nrf1 is constitutively retrotranslocated from the ER lumen to the cytoplasm, where it is degraded by the proteasome under homeostatic conditions [177,178,179]. However, if proteasome activity is inhibited or hindered, then Nrf1 is instead cleaved by the DDI2 protease in the cytoplasm to form the active transcription factor that translocates to the nucleus and enhances transcription of UPS genes, among others [180,181]. This allows for a “bounce-back” response in which cells react to proteasome inhibition by producing more proteasome subunits. As an aspartyl protease with a substrate binding site, DDI2 might be a promising, potentially “druggable” candidate for combination therapies with proteasome inhibitors. However, for this to be a viable strategy, demonstration is critical of the requirement of DDI2 and Nrf1 for this compensatory mechanism in actual human-cancer-patient samples. Additionally, more thorough characterization of the DDI2 cleavage mechanism, including reconstitution of the activity of the human protein and characterization of its other roles in the cell, are critical. In addition to DDI2, Nrf1 deglycosylation by the NGLY1 enzyme was demonstrated to be important for Nrf1 activity [182,183]. Although loss of NGLY1 is responsible for a rare human genetic disorder, and the range of its substrates necessitate thorough characterization [184], inhibiting NGLY1 might serve as another druggable target in this pathway.

An additional metabolic mechanism of resistance was recently described and can potentially be exploited therapeutically. The mechanism involves a surprising relationship between proteasome inhibition and mitochondrial metabolism [185]. Resistance to proteasome inhibitors is related to a shift from glycolysis to oxidative phosphorylation, and such a shift renders cells especially sensitive to the small molecule elesclomol, whose target is the mitochondrial reductase FDX1. This finding raises the possibility that perhaps there are other resistance pathways that have not yet been discovered that can be exploited for therapeutic intervention.

Cumulatively, understanding mechanisms of resistance to proteasome inhibitors, and how to target them using combination therapies can help improve the efficacy of treatments for MM and MCL, and can potentially broaden the scope of proteasome inhibitors to the treatment of solid tumors—a holy grail. By increasing the extent and duration of proteasome inhibition by concomitantly inhibiting a resistance mechanism, cancer cells on the brink of apoptosis might be pushed over the edge [89].

### 3.2. Other UPS Inhibitors

#### 3.2.1. VCP/p97 Inhibition

There are many UPS components other than the 20S CP that can serve as viable targets for therapeutic intervention. One very promising oncological target is vasolisin-containing protein (VCP)/p97, an essential and conserved homohexameric AAA+ ATPase that has been demonstrated in recent years to be involved in numerous cellular pathways [186]. p97 is central to proteostasis by serving as a “segragase” that extracts challenging UPS substrates from cellular structures (downstream of E3 ubiquitin ligases) [187,188]. For example, p97 is essential for extracting ER-associated substrates during ERAD and tRNA-nascent chains from ribosomes that stall during translation [189]; p97 also separates protein aggregates so that the proteasome can degrade them. There are also nondegradative roles for p97, such as in endosomal trafficking and chromatin remodeling [190]. There have been substantial efforts to understand the structure and mechanism of p97, and the nature of its interaction with various adapter proteins that are required for its function in different pathways [191,192,193,194]. Because p97 is essential, it cannot be completely eliminated from cells without inducing the UPR and apoptosis. However, cancer cells appear to have a heightened dependence on p97 than noncancerous cells do, and hence more rapid apoptosis upon p97 depletion [89].

There have been substantial efforts to find effective and potent p97 inhibitors, and despite many tool compounds that can be used in the lab, there has been only one that made it to phase 1 clinical trials: CB-5083 developed by Cleave Biosciences. CB-5083 is an orally available, potent inhibitor that shows efficacy in tumor models that are insensitive to proteasome inhibitors. Additionally, CB-5083 inhibition is more efficacious in solid tumor models than proteasome inhibitors. Transcriptomics studies with MM cell lines indicate that the cellular response to CB-5083 is distinct from that of proteasome inhibitors, supporting the notion that targeting different components of the UPS can be beneficial for treating different diseases or disease states [195]. Interestingly, the transcription factor Nrf1 is an ERAD substrate and depends on p97 for its retrotranslocation [179]. CB-5083 inhibits Nrf1-mediated proteasome gene upregulation, thereby inhibiting a potentially important form of resistance to proteasome inhibition. While the pre-clinical data were promising for this first-in-class compound, clinical trials for its efficacy in treating MM were terminated in 2018.

P97 inhibition can be effective in treating diseases other than cancer. Inclusion body myopathy with early-onset Paget disease and frontotemporal dementia (IBMPFD) is caused by mutations in VCP [196,197]. Studies from a biochemical reconstitution of p97 demonstrate that IBMPFD mutants show enhanced p97 unfoldase activity [198,199]. Therefore, it is possible that inhibiting p97 activity can be efficacious for treating IBMPFD.

#### 3.2.2. Inhibiting other UPS Components

Despite the diversity and the number of E3 ubiquitin ligases, which confer substrate specificity to the ubiquitination reaction, there are only two mammalian E1 ubiquitin-activating enzymes. Theoretically, inhibiting the E1 reaction would inhibit all ubiquitin-dependent pathways in cells, both degradative and non-degradative. The first E1 inhibitor, PYR-41, was shown to attenuate NF-kB activation and proteasomal degradation of IkB. Proof-of-principle studies also demonstrated that it can differentially kill cancer cells [200]. A more potent and selective inhibitor, TAK-243, was recently reported to cause complete loss of cellular ubiquitylation, impaired cell cycle progression, and impaired DNA damage repair [201]. TAK-243 shows efficacy in tumor models and is under investigation as a clinical candidate for treating myelodysplastic syndrome and leukemia.

Targeting E3 ubiquitin ligases could be efficacious for finding more specific and less toxic inhibitors of the UPS. There are over 600 E3 ligases in mammalian cells that catalyze a range of ubiquitin linkages and chain types. Ubiquitinated proteins can have both degradative and nondegradative fates. The three main families of ligases, RING, U-box, and HECT-containing E3s, have different characteristics and properties. Most efforts to find E3 inhibitors have focused on MDM2, IAP, and SCF [202]. The IAP (antiapoptotic proteins) family inhibits proapoptotic caspases and Smac proteins. One target of IAP is RIP1, which activates NF-kB. Therefore, inhibiting RIP1 ubiquitylation inhibits NF-kB activation, and this has been exploited as a potential therapeutic strategy [203,204,205].

An indirect approach for inhibiting cullin-RING ubiquitin ligases, the largest class of E3 ligases, is to inhibit their activation by neddylation, post-translational modification similar to ubiquitylation. Pevonedistat (MLN4924/TAK-924) is a potent inhibitor of the Nedd8-activating enzyme (similar to the ubiquitin-activating E1 enzyme) that has shown efficacy in killing a variety of cancer-cell types, and has been under clinical investigation for treating leukemia in addition to solid tumors [206,207,208].

As described above, inhibiting Rpn11, the de-ubiquitinating (DUB) enzyme in the lid of the 19S RP, has proven efficacious in the lab. DUBs in general are promising targets for cancer therapies because they are aberrantly activated or expressed in certain cancer types. A challenge with identifying potent and selective DUB inhibitors is that DUBs contain an active-site cysteine, and many DUB inhibitors that have been discovered and investigated are thiol-reactive molecules. Therefore, they have the potential problem of reacting nonspecifically with exposed cysteine residues in various proteins. One way to circumvent this problem and to confer specificity is to have multiple binding modes or binding sites distal to the catalytic cysteine. This has been exploited for finding inhibitors of the DUB Usp7, for example, which show efficacy in tumor models [209,210,211,212]. There is precedent for bringing thiol-reactive drugs to the clinic [213,214,215,216,217]. The recent development of AMG 510 by Amgen for treating solid tumors with a particular KRAS-G12C mutation further demonstrates that cysteine-reactive drugs can advance to clinical trials [218].

Besides Rpn11, there are two proteasomal non-JAMM-domain-containing DUBs, Usp14 and Uch37 [8,219]. Interestingly, the activities of these DUBs appear to reduce degradation of their substrates, presumably because they are not coupled to the ATPase activity of the 19S RP. Consistent with this activity, a well-characterized small-molecular inhibitor of Usp14, IU1, showed increased degradation of model substrates, biochemically and in cells [219,220]. Recent structural evidence demonstrated that IU1 is specific for Usp14 by binding to an allosteric site that blocks access of ubiquitin to the active site [221]. This class of inhibitor could be useful for treating diseases in which proteasome activity needs to be activated, such as neurodegenerative diseases. Interestingly, b-AP15, a dual inhibitor of Usp14 and Uch37, was shown to prevent protein degradation and inhibit tumor progression in acute myeloid leukemia models [222].

### 3.3. Other Indications for Proteasome Inhibitors

Although clinical proteasome inhibitors were originally developed to treat cancers, the rich biology of the proteasome and its essentiality in many processes make it a promising target for other indications, too. For example, there is a straightforward connection between proteasome inhibition and immune disorders. The proteasome is involved in generating antigens for presentation by MHC Class I, and proteasome inhibitors are effective at targeting antibody-producing B cells. As such, bortezomib and carfilzomib continue to be explored as therapies for antibody-mediated allograft rejection (AMR) [223,224,225]. Proteasome inhibitors might be useful for treating AMR because they reduce the number of human leukocyte antigen (HLA) antibodies [226]. Bortezomib showed early efficacy when used alone according to the MM treatment regimen to treat rejection following renal transplantation [227]. Bortezomib has also been used in combination with other AMR treatments [228]. Additionally, proteasome inhibitors have been efficacious in treating heart, pancreas, and lung allograft rejection [229,230,231]. There are also clinical trials that were completed or are underway to evaluate the use of proteasome inhibitors to treat graft-versus-host disease and other transplantation-related immune disorders (clinicaltrials.gov).

Another interesting clinical application of proteasome inhibition is in the treatment of malaria [232,233,234,235]. Malaria parasites contain their own form of the 26S proteasome in the cytoplasm and nucleus. Additionally, in silico predictions and biochemical analyses suggest that a significant portion of the *Plasmodium falciparum* proteome can be ubiquitinated during asexual reproduction, and ubiquitination is associated with resistance to antimalarial agents [236]. Selective inhibitors of the protozoal proteasome have proven effective in killing *P. falciparum* while sparing human cells [237,238]. Because the proteasome is vital in all stages of the *P. falciparum* life cycle in human hosts, it remains a promising antimalarial drug target that merits further investigation.

While the protozoan proteasome is an unusual drug target, even more surprising is the use of proteasome inhibitors as antibiotics. Although the proteasome is essential in eukaryotes, there are conditionally essential proteasomal genes in many bacterial *Actinomycetales* and *Nitrospirales* lineages [239]. This includes highly pathogenic species *Mycobacterium tuberculosis*, a major global threat affecting at least 10 million people in 2017 [240,241,242,243,244]. Researchers have turned to the *M. tuberculosis* proteasome as a potential drug target because of its importance for resistance to oxidative and nitrosative stresses in a human host. As with malarial proteasome inhibitors, molecules targeting the *M. tuberculosis* proteasome must be selective over human proteasomes. Significant efforts were made to identify selective *M. tuberculosis* proteasome inhibitors [245,246], and with growing interest in using proteasome inhibitors to treat infectious diseases amid the threat of drug resistance, this is an exciting time to investigate fundamental properties of bacterial proteasomes and novel ways to distinguish them from human proteasome complexes [246].

## 4. Conclusions

**Figure 4:**
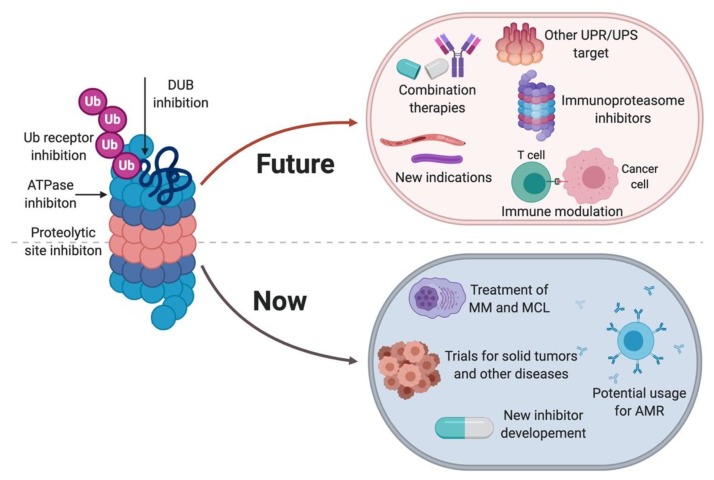
State of proteasome inhibitors, including summary of current state and future of proteasome inhibitors [85].

Proteasome inhibitors have proven efficacious in the clinic for treating hematological malignancies. Their efficacy for treating these cancer types and for expanding their indications to solid tumors has been challenged by the development of resistance and toxicity, in addition to past limitations in crossing the blood-brain barrier and therefore treating glioblastomas. However, innovative applications of proteasome inhibitors that can have clinical relevance continue to be uncovered. Considering the importance of the proteasome for antigen presentation by immune cells, uses for proteasome inhibitors (especially at low doses) in modulating antigen presentation and immunoproteasome-specific inhibitors will clearly be active areas of basic and applied research in the coming years. Exploring specific degradation trajectories of target proteins can also prove to be a viable approach for finding specific inhibitors, as there is tremendous diversity in the players involved in the UPS. Finding the right balance between inhibiting the proteasome “just enough” and maintaining proteasome activity where it is needed will continue to be a challenge that will need to be addressed. However, fundamental and applied research pertaining to the proteasome and proteasome inhibitors, and ongoing and new clinical trials that make use of proteasome inhibitors for treating a growing number of diseases, will inform future drug discovery efforts. The proteotoxic crisis exists in many cell types and is central to a vast number of human diseases, and as long as this is the case, there will be a need for more specific and selective proteasome inhibitors (Figure 4).

## Figures and Tables

**Table 1:** Proteasome inhibitors in the clinic and in clinical trials.

Name	Type	IC_50_ (nM)			β5 Dissoc. *T*_1/2_ (min)	Administration
		β5 Chymotrypsin-Like	β1 Caspase- Like	β2 Trypsin- Like		
Bortezomib (PS-341)	Boronate	2.4–7 *^,#^	24–74 *^,#^	1200–4200 *^,#^	110 ^#^	Intravenous
Carfilzomib (PR-171)	Epoxyketone	6 *	2400 *	3600 *	Irreversible	Intravenous
Ixazomib (MLN9708)	Boronate	3.4^#^	31 ^#^	3500 ^#^	18 ^#^	Oral
Marizomib (Salinosporamide A)	β-lactone	3.5 *	430 *	28 *	Irreversible	Intravenous
Oprozomib (ONX0912)	Epoxyketone	36 ^^^	NA	NA	Irreversible	Oral

* [98]; ^#^ [105]; ^ [106].
